# The Essential Oil of *Monarda didyma* L. (Lamiaceae) Exerts Phytotoxic Activity In Vitro against Various Weed Seeds

**DOI:** 10.3390/molecules22020222

**Published:** 2017-02-02

**Authors:** Donata Ricci, Francesco Epifano, Daniele Fraternale

**Affiliations:** 1 Dipartimento di Scienze Biomolecolari, Università degli Studi di Urbino Carlo Bo, sez. Biologia Vegetale, via Bramante 28 61029 Urbino (PU), Italy; donata.ricci@uniurb.it; 2 Dipartimento di Farmacia, Università degli Studi di Chieti-Pescara G. d’Annunzio, via dei Vestini 31 66100 Chieti (CH), Italy; francesco.epifano@unich.it

**Keywords:** essential oil, anti-germination, *Monarda didyma* L., hydrogen peroxide, malondialdehyde, phytotoxic activity

## Abstract

The chemical composition of the essential oil of the flowering aerial parts of *Monarda didyma* L. cultivated in central Italy was analyzed by Gas Chromatography/Mass Spectrometry (GC/MS). The major compounds of the oil were thymol (59.3%), *p*-cymene (10.3%), terpinolene (9.2%), *δ*-3-carene (4.4%), myrcene (3.7%), and camphene (3.4%). The essential oil was tested in vitro for its anti-germination activity against *Papaver rhoeas* L., *Taraxacum officinale* F. H. Wigg., *Avena fatua* L., *Raphanus sativus* L. and *Lepidium sativum* L. seeds, demonstrating good inhibitory activity in a dose-dependent way. The exposure of the employed weed seeds to *M. didyma* essential oil and thymol solution (59.3%) increased the level of hydrogen peroxide (H_2_O_2_) and malondialdehyde (MDA), markers of oxidative stress, in emerging 5-day-old rootlets.

## 1. Introduction

*Monarda didyma* L. (golden balm or honey balm) grows spontaneously in North America and belongs to the Lamiaceae family; it is also cultivated in Europe. Its flowers are used to prepare an infusion called ‘the rouge’ or ‘Oswego tea’ in folk medicine, particularly used in the treatment of digestive disorders [1].

The following pharmacological properties have been attributed to the leaves and flowering stems; antihelmintic, carminative, diuretic, expectorant, febrifuge, rubefacient, and stimulant [2].

The essential oils of various parts of the *M. didyma* plant have been studied, as well as their antimicrobial and antioxidant activities [1,2,3,4,5,6].

Very recently, a protocol for the production of *M. didyma* and *M. fistulosa* essential oils has been developed. These oils are richer in thymoquinone and thymohydroquinone, which are believed to have anti-inflammatory effects with few side effects typical of known anti-inflammatories [7].

The essential oils from several plants and their components have been recently considered for their anti-germination activities against the seeds of several weeds [8,9,10]; this might be important in understanding the mechanisms of ecological interactions but also in identifying extracts or pure compounds for the formulation of natural products for agricultural use with low or no environmental impact.

Today there is a growing interest in natural products that are able to control the growth of weeds; in fact, the extensive use of synthetic herbicides has resulted in herbicide resistant weeds and risks for human and animal health because an intensive use of synthetic herbicides at high concentrations also increases the risk of toxic residues in agricultural products. Natural compounds derived from plants show a short half-life because they are easily biodegradable and thus do not produce or produce little damage to the environment [11].

The purpose of our study was to analyze the composition of the essential oil extracted from *M. didyma* plants cultivated in central Italy and to evaluate its anti-germination activity; this biological activity, to the best of our knowledge, is not yet reported in the literature for this essential oil.

Furthermore, it has been reported that the potential anti-germination and herbicidal activities possessed by some essential oils may also be related to the pro-oxidant activity that these natural compounds exert on germinating seeds and seedlings [12,13]. Therefore, another goal of our work was to show if the essential oil of *M. didyma*, and perhaps its major constituent (thymol), are able to induce oxidative stress in the germinating seeds used by us.

## 2. Results and Discussion

### 2.1. Chemical Composition of Essential Oil

Nineteen compounds were identified in the essential oil obtained from *M. didyma*, with flowering aerial parts accounting for 99.8% of the total volatiles. These are listed in Table 1. The main components were oxygenated monoterpenes (60.4%), followed by monoterpene hydrocarbons (34.7%), sesquiterpene hydrocarbons (2.1%), and, lastly, 1-octen-3-ol (C_8_H_16_O 2.5%), a secondary alcohol derived from 1-octene. Thymol was the main constituent (59.3%). The other representative compounds were *p*-cymene (10.3%), terpinolene (9.2%), δ-3-carene (4.4%), myrcene (3.7%), and camphene (3.4%). The chemical composition of this essential oil obtained from plants grown at 67 m above sea level is very similar to that previously reported by us for a *M. didyma* essential oil extracted from plants grown at about 500 m above sea level [1] and is also in agreement with the analysis of an oil obtained from plants cultivated in France [14].

Thymol was also identified as the main constituent in the *M. didyma* oil from plants grown in western Siberia and Lithuania [1].

However, other studies on the composition of this essential oil show low thymol content or the absence of thymol and the presence of 1,8-cineole as the main constituent, as reported previously by us [1].

### 2.2. Anti Germination Activity

In this study, the *M. didyma* essential oil was evaluated for its phytotoxic activity against germination and the initial radical elongation of red poppies (*Papaver rhoeas* L.); dandelions (*Taraxacum officinale* F.H. Wigg.); wild oats (*Avena fatua* L.), considered a common weed; radish (*Raphanus sativus* L.); and garden cress (*Lepidium sativum* L.), of which two species are usually employed in these bioassays [8,9]. The oil had a different effect on the germination and root elongation of the five seeds used. Poppy seed germination was totally inhibited by the EO (Essential Oil), PCM (Pure Compounds Mixture), and 59.3% thymol solution at 1.250 μg/mL, while 0.250 μg/mL inhibited this parameter by about 50%. The solutions of *p*-cymene (10.3%) and terpinolene (9.2%) inhibited the germination of poppy seeds at the highest concentrations tested; about 70% and 60%, respectively (Table 2).

A concentration of 0.125 μg/mL of the EO and the PCM significantly inhibited the poppy seeds’ radical elongation; among the pure compounds, the thymol solution (59.3%) and the *p*-cymene solution (10.3%) also showed the same effect, while the terpinolene solution did not inhibit the root growth of poppy seeds (Table 3). The seed germination of *T. officinale*, an important medicinal species but also considered a weed in cultivated areas, was already inhibited by 0.125 μg/mL of the EO, PCM, and thymol solution (59.3%). The solution of terpinolene (9.2%), at any concentration tested, did not inhibit this germination. A concentration of 1.250 μg/mL of the EO, PCM, or thymol solution (59.3%) totally inhibits the in vitro germination of dandelion seeds (Table 2). Considering the effect of the essential oil of *M. didyma* on the radical elongation of germinated dandelion seeds, 0.250 μg/mL was sufficient to induce an inhibition of about 40% compared to the control, together with browning of the root and necrosis of the root tips (Table 3). The germination of *A. fatua* caryopsis was inhibited by the EO, PCM, and thymol solution (59.3%) by about 80% at 1.250 μg/mL; the *p*-cymene solution (10.3%) and the terpinolene solution (9.2%) were less active, inducing an anti-germination effect of about 60%, compared with the control (Table 2). The EO, PCM, and thymol solution at 0.125 μg/mL already inhibit the radical elongation of *A. fatua* caryopsis by about 60%, while *p*-cymene solution (10.3%) and terpinolene solution (9.2%) inhibit it at 0.250 μg/mL (Table 3).

In all the tests, the concentration of 0.625 μg/mL for all the seeds employed produces root browning and necrosis of the root tips; this is certainly a stress index that can lead to a growth delay or lack of development of the seedlings. All concentrations tested showed no inhibition of *R. sativus* seed germination, while the 0.06 μg/mL concentrations of the EO, PCM, and thymol solution significantly inhibited radical elongation in radishes. Furthermore, already at a 0.250 μg/mL concentration, the EO, PCM and thymol solution induce browning and necrosis of root tips. On the contrary, the solution of *p*-cymene and terpinolene does not induce any effect on *R. sativus* root growth (Table 2 and Table 3). Concerning *L. sativum*, only the thymol solution at 0.625 μg/mL inhibited seed germination by about 35%, while the EO and PCM were active at the highest concentration tested (1.250 μg/mL), inhibiting the germination of garden cress seeds by approximately 50% (Table 2).

The radical elongation of germinated *L. sativum* seeds was inhibited by the EO, PCM, and thymol solution, starting from 0.625 μg/mL. 1.250 μg/mL also produces browning of apexes and quick death of the roots (Table 3).

The results obtained show that, in general, the inhibitory effects of the *M. didyma* EO, PCM, and the solution of pure compounds tested on seed germination and root elongation increased with the increase in the concentration employed. Regarding germination inhibition, dandelion seeds are the most sensitive seeds because they are inhibited by 25% at 0.125 μg/mL concentrations of the EO, PCM, and thymol solution. The most resistant seeds to the anti germination action of *M. didyma* essential oil were those of *R. sativus*, insensitive at all concentrations tested. Concerning the effect on radical elongation, *P. rhoeas*, *T. officinale*. and *R. sativus* seeds were the most sensitive because 0.06 μg/mL of the EO, PCM, and thymol solution inhibited this parameter by about 25%, 28%, and 21%, respectively, while the radical elongation of *L. sativum* seeds was inhibited by about 39% at a 0.625 μg/mL concentration of the EO, PCM, and thymol solution.

Interestingly, 0.625 μg/mL and, to a greater extent, 1.250 μg/mL of the EO, PCM, and solutions of all pure compounds for *P. rhoeas*, *T. officinale*, and *A. fatua*, and only the thymol solution for *R. sativus* and *L. sativum* induces blackening, followed by necrosis of the roots, which induces the arrest of the development of seedlings in vitro.

The mechanism of the inhibitory action of essential oils remains unclear, although it has been reported that volatile oils, particularly monoterpenes, inhibit the cell division of apical meristems. Probably, the main cause of this action is the generation of Reactive Oxygen Species (ROS)—induced oxidative stress and the inhibition of DNA synthesis or disruption of the membranes [12].

Several authors have shown that essential oils rich in phenolic compounds (thymol is a phenolic compound) possess interesting inhibitory effects on seed germination and the radical and seedling growth of several plants [15,16,17]. These essential oils may also induce oxidative stress during seed germination and seedling growth.

This condition of oxidative stress can be detected by an increase in H_2_O_2_ (hydrogen peroxide), a reactive oxygen species (ROS); we know that the increase in hydrogen peroxide is due to the increasing enzymatic action of superoxide dismutase (SOD), an enzyme of the intracellular antioxidant system, the activity of which increases in oxidative stress conditions [18]. A high level of ROS can cause damage to cell membranes, as well as lipid peroxidation, but also damage to DNA as well as protein and cell death [19].

**Figure 1:**
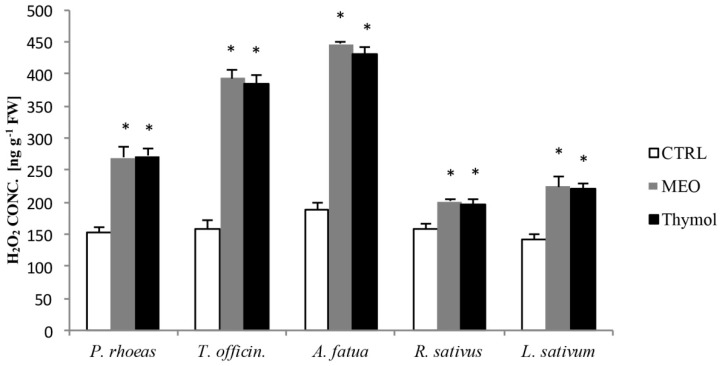
Hydrogen peroxide (H_2_O_2_) concentration in five-day-old rootlets of the examined weed seeds germinating in control (CTRL), *Monarda didyma* essential oil (MEO) at 0.625 μg/mL, and 59.3% thymol solution at 0.625 μg/mL. * *p* ≤ 0.01 vs CTRL, according to Student’s *t* test.

The essential oil of *M. didyma* and its main constituent thymol produce, at 0.625 μg/mL, a significant increase in the level of H_2_O_2_ in the roots, and this further increases the lipid peroxidation at five days of culture in all the five weed seeds tested; *P. rhoeas*, *T. officinale, A. fatua*, *R. sativus*, and *L. sativum*, (Figure 1).

The increase in lipid peroxidation can be shown through the increase in malondialdehyde (MDA), a product of unsaturated fatty acid peroxidation in phospholipids, responsible for cell membrane damage [11].

**Figure 2:**
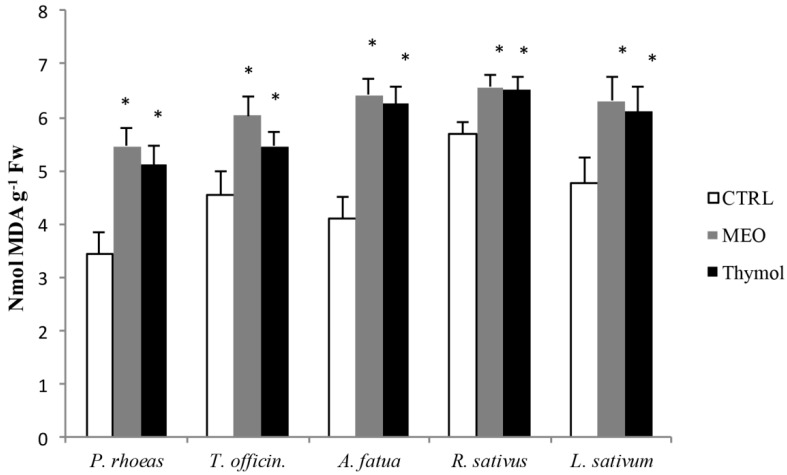
Malondialdehyde (MDA) content of THE five-day-old rootlets of the examined weed seeds germinating in control (CTRL), *Monarda didyma* essential oil (MEO) at 0.625 μg/mL, and 59.3% thymol solution at 0.625 μg/mL. * *p* ≤ 0.01 vs CTRL, according to Student’s *t* test.

In our experiment, simultaneous with the increase in hydrogen peroxide caused by the essential oil from *M. didyma* and by the thymol, we also observed an increase in MDA content and, consequently, an increase in the lipid peroxidation in the roots of all the seeds tested. This could be one reason for the blackening and then necrosis of the roots in our experiment (Figure 2).

The essential oil from Turkish *Origanum acutidens* (Hand.-Mazz.) and its two main components, carvacrol and thymol , were shown to possess phytotoxic activity against the seed germination and seedling growth of *Amaranthus retroflexus* L., *Chenopodium album* L., and *Rumex crispus* L.; the authors also hypothesized a mechanism based on membrane damage as a result of a pro-oxidant effect [16]. In our paper, we confirm that the thymol exerts phytotoxic activity against the seeds used but also that this activity may be due to an increase in oxidative stress in the tissues of rootlets.

To our knowledge, other essential oils such as the essential oil of *Nepeta meyeri* Benth, increased the level of hydrogen peroxide and lipid peroxidation in several weeds [8], but, for the first time, this ability has been revealed for the essential oil of *M. didyma* and for thymol, its main constituent.

The phytotoxic activity of *p*-cymene was evaluated for the first time by [16]; vapors of *p*-cymene inhibited the seed germination of *C. album* and *R. crispus* while they weakly suppressed the germination of *A. retroflexus* seeds. Successively, *p*-cymene showed no effect on the germination and root elongation of *R. sativus* and *L. sativum* [8]. In this paper, we confirm the data reported by [8], but we have also shown that a 10.3% solution of *p*-cymene, at 1,250 μg/mL, completely inhibits the germination of *P. rhoeas* and *T. officinale* seeds, while it inhibits the germination of *A. fatua* by 57% and the elongation of the roots of this caryopsis by 85% (Table 2 and Table 3). For the first time, to our knowledge, in this work terpinolene was tested to evaluate its possible phytotoxicity; this partially inhibits the germination and root elongation of *P. rhoeas*, *T. officinale*, and *A. fatua*, while it showed no phytotoxic effects on *R. sativus* and *L. sativum* seeds, as shown in Table 2 and Table 3. However, it was reported that the essential oil of *Ferulago angulata* (Schlecht.) Boiss., which contains γ-terpinolene as a main component (11.97%), exhibits considerable phytotoxic activity [20].

The essential oil we used is mostly composed of hydrocarbon and oxygenated monoterpenes. The literature reports that the essential oils rich in monoterpenes are more active, as regards phytotoxic activity, than essential oils with a high percentage of sesquiterpenes and a low monoterpene content [21,22]. The monoterpenes are lipophilic and cross the cell membrane, causing damage to its structure and to membrane enzymes’ this, together with the eventual pro-oxidant activity, could represent the mechanism by which some essential oils result in phytotoxicity [8].

## 3. Experimental

### 3.1. Plant Material

The *M. didyma* plants employed for the essential oil extraction were grown in an experimental field located at ITAC (Istituto Tecnico Agrario e Chimico - Agricultural Technical and Chemical Institute), Imola (BO), Italy (67 m above sea level). The plants’ flowering aerial parts were harvested in October 2013; a specimen of the plant has been deposited in the herbarium of the ITAC (Md 19-63).

The monoterpene standards were purchased from: thymol (w306606 Sigma-Aldrich, Darmstadt, Germany, ≥99%), p-cimene (C12,145-2 Aldrich, 99%), and terpinolene (86485 Fluka, ≥85%).

### 3.2. Essential Oil Isolation

The plant material was steam distilled in a Clevenger-type apparatus for 3 h until the material was exhausted, with a yield (*v*/*w*) of 2.61 mL/kg dry weight.

The oil was dried over anhydrous sodium sulfate and, after filtration, stored at +4 °C until analyzed and used in biological tests.

### 3.3. Essential Oil Analyses

The oils were analyzed by GC and GC/MS. Analyses were performed on a Hewlett-Packard gas chromatograph, model 5890, equipped with a Flame Ionization Detector (FID) and coupled to an electronic integrator. The chromatograph was fitted with methyl silicone column (MetSil) (12.5 m × 0.25 mm, 0.25 µm film thickness). The carrier gas was helium (0.9 mL/min); the injector and detector temperatures were 280 °C and 250 °C, respectively. The oven temperature was programmed from 50 °C to 270 °C at a rate of 4 °C/min. Quantitative data were obtained by electronic integration of FID area data without the use of response factor correction. GC/MS analyses were performed using a Hewlett-Packard 6890 chromatograph combined with HP Chem Station software, equipped with a 5973 mass selective detector, and mass spectrometer was operated at 70 eV, with a scanning speed of 1 s over a 40–300 amu range and an ion source temperature of 180 °C. Compounds were identified by comparison of the GC retention indices relative to the retention times of a series of n-alkanes (C7-C25) with those reported in the literature [23] and by a comparison of mass spectra from the Nist98 Mass Spectral Database. GC analyses using a polar column were carried out on a Varian 3400 GC system, equipped with a fused silica CP-Sil 88 column (50 × 0.22 mm, 0.2 μm film thickness). The carrier gas was helium (0.9 mL/min). The oven temperature was programmed from 50 °C to 270 °C at a rate of 4 °C/min [1]. Component identification was carried out by a comparison of calculated retention indices with those reported in the literature [23].

### 3.4. Biological Assay

The *M. didyma* EO anti germination activity was tested following the method already used by us [24]. We tested the following pure compounds potentially responsible for phytotoxic activity, present in *M. didyma* essential oil (EO); thymol, *p*-cymene, and terpinolene (all Sigma-Aldrich, Darmstadt, Germany). The three solutions were prepared so that each pure compound was present in the same percentage as that in the original *M. didyma* EO; 59.3%, 10.3%, and 9.2%, respectively, in H_2_O/Acetone (99.5/0.5) [8]. A Pure Compounds Mixture (PCM) was also prepared by mixing in H_2_O/Acetone solution, as above, the three pure compounds in the same percentages present in the EO. The PCM and each solution of the pure compound were used in the tests for seed-germination at the same concentrations used for the original EO; 0.06, 0.125, 0.250, 0.625, and 1.250 μg/mL. The seeds of *R. sativus*, *L. sativum* L., and *P. rhoeas* were purchased from Fioral srl, Cesena (FC), Italy. The seeds of *A. fatua* and *T. officinale* were collected from wild plants and identified by Dr. Daniele Fraternale. The seeds were surface cleaned in 95% ethanol for 15 s, rinsed three times in distilled water, and sown in Petri dishes (diameter = 90 mm) containing 5 layers of Whatman filter paper, soaked with 7 mL of either distilled water (control), the EO, the PCM, or the solutions of the major pure components found in the EO. The culture conditions were 24 ± 1 °C with a 16 h photoperiod of 1500 lux light. The herbicide 2,4-D Isooctylester (Sigma-Aldrich, Darmstadt, Germany) to a concentration of 0.06 μg/mL was also used as reference. The acetone-water mixture behaves like water alone against the germination and radical elongation of the seeds used as control. Each Petri dish contained 10 seeds. A seed was considered germinated when the protrusion of the radicle became evident. The number of germinated seeds and the length of the roots were recorded after five days [8]. Data are expressed as the mean ± SD of both germination and radical elongation. Each experiment was made in triplicate.

### 3.5. Determination of Hydrogen Peroxide Concentration

The concentrations of hydrogen peroxide in five-day-old roots were determined according to [25], by measuring the titanium peroxide complex absorbance at 450 nm. One g of roots material was extracted in 3 mL of ice-cold acetone, and 1 mL of extract was added to 0.1 mL of 20% titanium reagent and 0.2 mL ammonia solution with 17 mol. After the centrifugation of this solution at 3000*× g* at 4 °C for 10 min, the supernatant was discarded and the pellet was dissolved in 3 mL of sulfuric acid, with 1 mol. The solution absorbance was measured at 410 nm with a ‘Carlo Erba reagents’ spectrophotometer 6305. A standard curve, obtained with known concentrations of hydrogen peroxide, was used to calibrate the absorbance values read in the different conditions.

### 3.6. Determination of Lipid Peroxidation

The lipid peroxidation level in five-day-old roots was measured with the method indicated by [11]. One g of the roots grown under different conditions was homogenized in 3 mL of 0.1% TCA (trichloroacetic acid) and centrifuged at 15,000× *g* for 30 min at 4 °C. One mL of the reagent obtained by mixing 0.5% thiobarbituric acid (TBA) in 20% TCA was added to 0.5 mL of previously obtained supernatants.

A negative control was created by mixing 0.5 mL of 0.1% TCA and 1 mL of reagent, obtained as above.

The test tubes were heated at 95 °C for 30 min and then quickly cooled in an ice bath; after centrifugation, the supernatant absorbance was read at 532 nm, and the value for the non-specific absorption at 600 nm was subtracted. The mmol/L extinction coefficient of 155 mmol/(1 cm) was used to assess the level of malondialdehyde (MDA).

### 3.7. Statistical Analysis

Student’s t test of independence was applied in all experiments. In the tests of radical elongation for non-germinated seeds, the length of the root was considered equal to 0 cm.

## 4. Conclusions

Based on our data, we think that *M. didyma* essential oil has the potential to be used as a constituent in the formulation of bio-herbicides. In future research, we will try to understand whether such activity, shown in vitro for the essential oil of *M. didyma*, is also maintained in the field.

## Figures and Tables

**Table 1:** Chemical composition of *Monarda didyma* essential oil from Imola (Bologna) (Italy).

Compounds	%	R_I_^a^	R_II_^b^
α-Pinene	0.8	942	1036
Camphene	3.4	952	1066
Myrcene	3.7	988	1142
δ-3-Carene	4.4	1008	1156
α-Phellandrene	1.1	1010	1175
*p*-Cymene	10.3	1016	1255
Limonene	1.4	1027	1187
β-Phellandrene	0.2	1028	1215
Terpinolene	9.2	1056	1247
1-Octen-3-ol	2.5	1087	1522
Linalool	0.7	1102	1506
α-Terpineol	0.1	1186	1731
Thymol methyl ether	0.2	1259	1735
Thymol	59.3	1269	1739
α-Copaene	0.3	1377	1551
β-Bourbonene	0.1	1385	1586
α-Humulene	0.2	1427	1522
Epi-Bicyclosesquiphel-landrene	0.2	1469	1524
Germacrene D	1.1	1488	1712
Total	99.8		
Monoterpene hydroc.	34.8		
Oxygenated monoterp.	60.4		
Sesquiterpene hydroc.	2.1		
Non terpenes	2.5		

^a^ = retention indices on MetSil column; ^b^ = retention indices on CP-Sil 88 column.

**Table 2:** Anti-germination effects (the average of germinated seeds for three replications ± SD) of EO, PCM, and solutions of the three major compounds of *Monarda didyma* essential oil at the same percentage found in the oil and 2,4-D Isooctylester against the germination of *Papaver rhoeas*, *Taraxacum officinale*, *Avena fatua*, *Raphanus sativus*, and *Lepidium sativum* five days after sowing. Results are the mean of three experiments ± SD.

Plant	Samples/conc.	0.06 μg/mL	0.125 μg/mL	0.250 μg/mL	0.625 μg/mL	1.250 μg/mL
*P.rhoeas*	EO	10 ± 0.0				
	PCM	10 ± 0.0	8.6 ± 0.5	5.3 ± 1.5 *	2.6 ± 0.5 **	0.0 ± 0.0
	Thymol/59.3%	9.3 ± 1.1	8.0 ± 1.0	5.0 ± 1.0 *	2.6 ± 1.1 **	0.0 ± 0.0
	*p*-Cymene/10.3%	10 ± 0.0	7.6 ± 1.1	5.3 ± 0.5 **	3.0 ± 1.7 *	0.0 ± 0.0
	Terpinolene/9.2%	10 ± 0.0	9.3 ± 0.5	7.6 ± 1.5	5.0 ± 1.0 *	3.3 ± 1.5 *
	2,4-D Isooctylester	0.0 ± 0.0	9.0 ± 1.4	7.6 ± 0.5 *	4.6 ± 1.5 *	4.0 ± 1.0 *
	Control (H_2_O)	10 ± 0.0				
*T. officin.*	EO	10 ± 0.0				
	PCM	10 ± 0.0	8.3 ± 0.5*	4.6 ± 1.5 *	1.6 ± 1.5 *	0.0 ± 0.0
	Thymol/59.3%	10 ± 0.0	7.0 ± 1.0*	5.6 ± 1.1 *	1.6 ± 1.1 **	0.0 ± 0.0
	*p*-Cymene/10.3%	10 ± 0.0	7.3 ± 1.1*	5.1 ± 1.7 *	1.3 ± 0.5 **	0.0 ± 0.0
	Terpinolene/9.2%	10 ± 0.0	9.3 ± 1.1	7.3 ± 0.5 *	7.6 ± 0.5 *	2.6 ± 1.5 *
	2,4-D Isooctylester	0.0 ± 0.0	9.0 ± 1.0	7.6 ± 1.1	7.3 ± 2.0	7.6 ± 0.5
	Control (H_2_O)	10 ± 0.0				
*A. fatua*	EO	10 ± 0.0				
	PCM	10 ± 0.0	10 ± 0.0	7.3 ± 0.5	4.3 ± 1.5 *	2.0 ± 1.0 **
	Thymol/59.3%	10 ± 0.0	10 ± 0.0	7.0 ± 1.0	5.0 ± 1.0 *	1.6 ± 0.5 **
	*p*-Cymene/10.3%	10 ± 0.0	10 ± 0.0	6.3 ± 0.5 *	3.6 ± 0.5 **	2.3 ± 0.5 **
	Terpinolene/9.2%	10 ± 0.0	10 ± 0.0	8.3 ± 0.5	6.6 ± 1.1 *	4.3 ± 1.1 *
	2,4-D Isooctylester	0.0 ± 0.0	10 ± 0.0	8.6 ± 1.5	7.6 ± 0.5 *	4.6 ± 1.5 *
	Control (H_2_O)	10 ± 0.0				
*R. sativus*	EO	10 ± 0.0				
	PCM	10 ± 0.0	10 ± 0.0	10 ± 0.0	9.0 ± 1.0	8.6 ± 0.6
	Thymol/59.3%	10 ± 0.0	10 ± 0.0	10 ± 0.0	8.6 ± 0.5	9.3 ± 0.6
	*p*-Cymene/10.3%	10 ± 0.0	10 ± 0.0	10 ± 0.0	10 ± 0.0	8.6 ± 1.1
	Terpinolene/9.2%	10 ± 0.0	10 ± 0.0	10 ± 0.0	10 ± 0.0	10 ± 0.0
	2,4-D Isooctylester	0.0 ± 0.0	10 ± 0.0	8.0 ± 1.0	8.0 ± 1.7	9.3 ± 1.1
	Control (H_2_O)	10 ± 0.0				
L. *sativum*	EO	10 ± 0.0				
	PCM	10 ± 0.0	8.3 ± 1.2	7.6 ± 1.1	6.3 ± 1.5	4.0 ± 1.0 *
	Thymol/59.3%	8.6 ± 0.4	8.6 ± 0.4	8.0 ± 1.0	6.3 ± 0.5	4.6 ± 0.5 *
	*p*-Cymene/10.3%	9.3 ± 0.9	8.6 ± 0.9	7.3 ± 0.5	6.0 ± 1.0 *	3.6 ± 1.1 *
	Terpinolene/9.2%	8.3 ± 0.4	10 ± 0.0	10 ± 0.0	10 ± 0.0	8.6 ± 0.4
	2,4-D Isooctylester	0.0 ± 0.0	9.3 ± 0.5	10 ± 0.0	10 ± 0.0	7.3 ± 0.9
	Control (H_2_O)	9.3 ± 1.1				

The values followed by * (* *p* < 0.05; ** *p* < 0.01 vs control) are statistically different according to the Student’s *t* test.

**Table 3:** Effects of radical elongation (cm) of EO, PCM, and solutions of the three major compounds of *Monarda didyma* essential oil at the same percentage found in the oil and 2,4-D Isooctylester against the germination of *Papaver rhoeas*, *Taraxacum officinale*, *Avena fatua*, *Raphanus sativus*, and *Lepidium sativum* five days after sowing. Results are the mean of three experiments ± SD.

Plant	Samples/conc.	0.06 μg/mL	0.125 μg/mL	0.250 μg/mL	0.625 μg/mL	1.250 μg/mL
*P.rhoeas*	EO	1.3 ± 0.2 **				
	PCM	1.2 ± 0.2 *	0.9 ± 0.2 *	0.6 ± 0.1 *	0.1 ± 0.05 **	0.0 ± 0.0 ***
	Thymol/59.3%	1.2 ± 0.05 *	1.0 ± 0.1 **	0.4 ± 0.1 **	0.1 ± 0.1 **	0.0 ± 0.0 ***
	*p*-Cymene/10.3%	1.4 ± 0.1	0.9 ± 0.2 *	0.4 ± 0.1 ***	0.2 ± 0.05 **	0.0 ± 0.0 ***
	Terpinolene/9.2%	1.5 ± 0.1	1.0 ± 0.1 *	0.6 ± 0.1 *	0.4 ± 0.1 **	0.1 ± 0.05 **
	2,4-D Isooctylester	0.0 ± 0.0	1.3 ± 0.05	1.4 ± 0.05	1.3 ± 0.2	0.8 ± 0.1
	Control (H_2_O)	1.6 ± 0.8				
*T. officin.*	EO	2.0 ± 0.2 *				
	PCM	2.0 ± 0.1 *	1.6 ± 0.1 **	1.1 ± 0.2 **	0.6 ± 0.2 **	0.0 ± 0.0 ***
	Thymol/59.3%	1.9 ± 0.2 *	1.4 ± 0.1 *	1.1 ± 0.1 **	0.6 ± 0.2 **	0.0 ± 0.0 ***
	*p*-Cymene/10.3%	2.1 ± 0.1 *	1.6 ± 0.2 ***	1.1 ± 0.2 *	0.5 ± 1.5 **	0.0 ± 0.0 ***
	Terpinolene/9.2%	2.1 ± 0.1	2.0 ± 0.1 **	1.7 ± 0.1 **	1.0 ± 1.7 **	0.3 ± 0.2 **
	2,4-D Isooctylester	0.0 ± 0.0	1.9 ± 0.3 *	1.7 ± 0.2	1.2 ± 0.1 **	0.3 ± 0.2 **
	Control (H_2_O)	2.8 ± 0.1				
*A. fatua*	EO	2.7 ± 0.2				
	PCM	2.7 ± 0.2	1.7 ± 0.2 **	0.8 ± 0.2 ***	0.4 ± 0.1***	0.1 ± 0.1***
	Thymol/59.3%	2.7 ± 0.2	1.7 ± 0.2*	0.7 ± 0.2 ***	0.4 ± 0.1**	0.1 ± 0.1***
	*p*-Cymene/10.3%	2.9 ± 0.1	1.6 ± 0.1**	0.6 ± 0.2 ***	0.3 ± 0.1***	0.1 ± 0.1***
	Terpinolene/9.2%	2.8 ± 0.1	2.2 ± 0.3	1.9 ± 0.1 **	1.0 ± 0.7**	0.4 ± 0.2***
	2,4-D Isooctylester	0.0 ± 0.0	2.4 ± 0.1	1.6 ± 0.1 **	0.9 ± 0.1***	0.3 ± 0.2***
	Control (H_2_O)	2.7 ± 0.1				
*R. sativus*	EO	2.9 ± 0.1 **				
	PCM	2.9 ± 0.05 *	2.7 ± 0.2 **	2.6 ± 0.1 *	2.0 ± 0.1 **	0.4 ± 0.2 **
	Thymol/59.3%	2.8 ± 0.1 *	2.7 ± 0.2 ***	2.4 ± 0.2 *	1.9 ± 0.05 **	0.3 ± 0.2 **
	*p*-Cymene/10.3%	3.7 ± 0.2	2.5 ± 0.1 *	2.1 ± 0.1 *	1.7 ± 0.1 **	0.2 ± 0.05 **
	Terpinolene/9.2%	3.3 ± 0.2	3.4 ± 0.3	3.2 ± 0.2	3.3 ± 0.3	3.4 ± 0.2
	2,4-D Isooctylester	0.0 ± 0.0	3.3 ± 0.2	3.2 ± 0.3	2.9 ± 0.1	3.1 ± 0.3
	Control (H_2_O)	3.7 ± 0.2				
*L. sativum*	EO	3.0 ± 0.1				
	PCM	2.9 ± 0.1	2.9 ± 0.2	3.1 ± 0.3	2.3 ± 0.2 *	1.5 ± 0.2 **
	Thymol/59.3%	3.1 ± 0.2	2.8 ± 0.3	3.0 ± 0.4	1.9 ± 0.1 *	1.2 ± 0.2 **
	*p*-Cymene/10.3%	3.1 ± 0.2	2.8 ± 0.2	2.7 ± 0.4	1.8 ± 0.1 *	1.5 ± 0.05 *
	Terpinolene/9.2%	3.0 ± 0.3	3.2 ± 0.1	3.0 ± 0.3	3.0 ± 0.4	2.7 ± 1.1
	2,4-D Isooctylester	0.0 ± 0.0	2.8 ± 0.4	3.1 ± 0.1	3.0 ± 0.2	3.1 ± 0.3
	Control (H_2_O)	3.3 ± 0.3				

The values followed by * (* *p* < 0.05; ** *p* < 0.01; *** *p* < 0.001 vs control) are statistically different according to the Student’s *t* test.
